# Supplementary material for: Self-Perceived Impact of COVID-19 Pandemic by Dental Students in Bucharest
Source: Int J Environ Res Public Health. 2021 May 14;18(10):5249. doi: 10.3390/ijerph18105249 (PMC8156800; doi:10.3390/ijerph18105249)
Supplement: Supplementary file 1 [file ijerph-18-05249-s001.zip › ijerph-1210692-supplementary.pdf]

Table S1. Questionnaire on the level of psychological and educational impact of the COVID-19 pandemic on dental students

Question	Abbreviation	Questions, Response Scale and Romanian Translations (italic)	
**Q1**	Age	Age	*Vârsta*
**Q2**	Sex	Sex	*Sexul*
		1. Male<br>2. Female<br>3. Other	*1. Masculin*<br>*2. Feminin*<br>*3. Alul*
**Q3**	Leaving alone	You live alone?	*Locuiți singur?*
		1. Yes<br>2. No	*1. Da*<br>*2. Nu*
**Q4**	Year of study	Year of study	*Anul de studiu*
		1. I<br>2. II<br>3. III<br>4. IV<br>5. V<br>6. VI	
**Q5**	Level of stress	How do you assess the level of stress you feel as a result of the COVID-19 pandemic?	*Cum evaluați nivelul de stres pe care îl simțiți ca urmare a pandemiei COVID-19?*
		1. Non - existent<br>2. Very low<br>3. Low<br>4. High<br>5. Very high	*1. Inexistent*<br>*2. Foarte scăzut*<br>*3. Scăzut*<br>*4. Crescut*<br>*5. Foarte crescut*
**Q6**	Anxiety	How do you assess the anxiety feeling related to a possible infection with SarsCov-2?	*Cum apreciați sentimentul de anxietate legat de o posibilă infecție cu SarsCov-2?*
		1. Non - existent<br>2. Very low<br>3. Low<br>4. High<br>5. Very high	*1. Inexistent*<br>*2. Foarte scăzut*<br>*3. Scăzut*<br>*4. Crescut*<br>*5. Foarte crescut*
**Q7**	Sleep quality	How do you assess the change in sleep quality during the COVID-19 pandemic?	*Cum apreciați modificarea calității somnului în timpul pandemiei de COVID-19?*
		1. Non-existent<br>2. Very low<br>3. Low<br>4. High<br>5. Very high	*1. Inexistent*<br>*2. Foarte scăzut*<br>*3. Scăzut*<br>*4. Crescut*<br>*5. Foarte crescut*
**Q8**	Study motivation	How do you appreciate the motivation for individual study compared to the period before the COVID-19 pandemic?	*Cum apreciați motivația pentru studiul individual în comparație cu perioada anterioară pandemiei COVID-19?*
		1. No affect<br>2. Minor affect<br>3. Neutral<br>4. Moderate affect<br>5. Major affect	*1. Neafectată*<br>*2. Minim afectată*<br>*3. Opinie neutra*<br>*4. Moderat afectată*<br>*5. Foarte afectată*

Question	Abbreviation	Questions, Response Scale and Romanian Translations (italic)	
**Q9**	Academic learning	How do you appreciate your own level of acquiring of academic information through the online teaching system?	*Cum apreciați, din punctul dvs. de vedere, insusirea informatiei academice prin intermediul sistemului de predare online?*
		1. Non - existent<br>2. Very low<br>3. Low<br>4. High<br>5. Very high	*1. Inexistenta*<br>*2. Foarte scăzuta*<br>*3. Scăzut*<br>*4. Crescuta*<br>*5. Foarte crescuta*
**Q10**	Academic teaching	How do you evaluate the efficiency of teachers in transmitting theoretical and practical information in online teaching courses?	*Cum evaluați eficiența cadrelor didactice în transmiterea informațiilor teoretice și practice în cursurile de predare online?*
		1. Very low<br>2. Low<br>3. Neutral<br>4. High<br>5. Very high	*1. Foarte scazuta*<br>*2. Scazuta*<br>*3. Opinie neutra*<br>*4. Crescuta*<br>*5. Foarte crescuta*
**Q11**	Time for individual study	How do you think the time allocated to the individual study has modified in your case, compared to the period before the COVID-19 pandemic?	*Cum credeți că s-a modificat timpul alocat studiului individual în cazul dvs., comparativ cu perioada anterioară pandemiei de COVID-19?*
		1. Not modified<br>2. Minor modified<br>3. Neutral<br>4. Moderate modified<br>5. Major modified	*1. Nemodificat*<br>*2. Minim modificat*<br>*3. Opinie neutră*<br>*4. Moderat modificat*<br>*5. Major modificat*
**Q12**	Gain of practical skills	In light of the COVID-19 pandemic, how much was your acquisition of practical skills regarding dental work affected, given that you normally would have been gained them from patients during traditional clinical activities?	*În lumina pandemiei de COVID-19, cum apreciati că va este afectata dobandirea abilitatilor practice, care ar fi fost învățate prin practica la pacient în timpul activităților clinice tradiționale?*
		1. No affect<br>2. Minor affect<br>3. Neutral<br>4. Moderate affect<br>5. Major affect	*1. Neafectată*<br>*2. Minim afectată*<br>*3. Opinie neutră*<br>*4. Moderat afectata*<br>*5. Major afectata*
**Q13**	Future professional perspective	How do you evaluate the impact of this period on the profession of dentist in the future?	*Cum evaluați impactul acestei perioade asupra profesiei de medic dentist în viitor?*
		1. Non-existent<br>2. Very low<br>3. Low<br>4. High<br>5. Very high	*1. Inexistent*<br>*2. Foarte scăzut*<br>*3. Scăzut*<br>*4. Crescut*<br>*5. Foarte crescut*
